# Acceptance of seasonal influenza vaccination and associated factors among pregnant women in the context of COVID-19 pandemic in China: a multi-center cross-sectional study based on health belief model

**DOI:** 10.1186/s12884-021-04224-3

**Published:** 2021-11-03

**Authors:** Ruitong Wang, Liyuan Tao, Na Han, Jihong Liu, Chuanxiang Yuan, Lixia Deng, Chunhua Han, Fenglan Sun, Liqun Chi, Min Liu, Jue Liu

**Affiliations:** 1 Department of Epidemiology and Biostatistics, School of Public Health, Peking University, No.38, Xueyuan Road, Haidian District, Beijing, 100191 China; 2 Research Center of Clinical Epidemiology, Peking University Third Hospital, No.49 Huayuan North Road, Haidian District, Beijing, 100083 China; 3 Tongzhou Maternal and Child Health Hospital, No.38 Yuqiao Middle Road, Tongzhou District, Beijing, 101100 China; 4 The Second Affiliated Hospital of Kunming Medical University, No. 374 Dianmian Avenue, Kunming, 650101 Yunnan Province China; 5 Qianjiang Maternal and Child Health Hospital, 122 Jiefang Road, Qianjiang City, 43100 Hubei Province China; 6 Qianjiang Central Hospital, No. 22 Zhanghua Middle Road, Qianjiang City, 433100 Hubei Province China; 7 Qujing Maternal and Child Health Hospital, No. 371 liaokuo South Road, Qilin District, Qujing City, 655000 Yunnan Province China; 8 Shexian Maternal and Child Health Hospital, No. 237 Zhenxing Road, shecheng Town, Shexian City, 056400 Hebei Province China; 9 Haidian Maternal and Child Health Hospital, No. 33 Haidian South Road, Haidian District, Beijing, 100080 China; 10 National Health Commission Key Laboratory of Reproductive Health, Peking University Health Science Center, No.38, Xueyuan Road, Haidian District, Beijing, 100191 China

**Keywords:** COVID-19, Influenza vaccination, Pregnant women, Acceptance, Associated factors

## Abstract

**Background:**

Seasonal influenza can circulate in parallel with coronavirus disease (COVID-19) in winter. In the context of COVID-19 pandemic, the risk of co-infection and the burden it poses on healthcare system calls for timely influenza vaccination among pregnant women, who are the priority population recommended for vaccination. We aimed to evaluate the acceptance of influenza vaccination and associated factors among pregnant women during COVID-19 pandemic, provide evidence to improve influenza vaccination among pregnant women, help reduce the risk of infection and alleviate the burden of healthcare system for co-infected patients.

**Methods:**

We conducted a multi-center cross-sectional study among pregnant women in China. Sociodemographic characteristics, health status, knowledge on influenza, attitude towards vaccination, and health beliefs were collected. Locally weighted scatterplot smoothing regression analysis was used to evaluate the trends in the acceptance of influenza vaccine. Logistic regression was applied to identify factors associated with vaccination acceptance.

**Results:**

The total acceptance rate was 76.5% (95%CI: 74.8–78.1%) among 2568 pregnant women enrolled. Only 8.3% of the participants had a history of seasonal influenza vaccination. In the logistic regression model, factors associated with the acceptance of influenza vaccine were western region, history of influenza vaccination, high knowledge of influenza infection and vaccination, high level of perceived susceptibility, perceived benefit, cues to action and low level of perceived barriers. Among 23.5% of the participants who had vaccine hesitancy, 48.0% of them were worried about side effect, 35.6% of them lacked confidence of vaccine safety.

**Conclusions:**

Our findings highlighted that tailored strategies and publicity for influenza vaccination in the context of COVID-19 pandemic are warranted to reduce pregnant women’s concerns, improve their knowledge, expand vaccine uptake and alleviate pressure for healthcare system.

**Supplementary Information:**

The online version contains supplementary material available at 10.1186/s12884-021-04224-3.

## Background

Coronavirus disease (COVID-19) is a severe acute respiratory disease which was declared a pandemic by the World Health Organization (WHO) on March 11, 2020 and has become a major global public health issue [1]. While the overall epidemic situation around the world have started to show signs of slowing down, countries continue to struggle with access to vaccines and the spread of emerging SARS-CoV-2 variants. By 15 June, 2021, there have been over 175 million cumulative cases and 3 million deaths reported around the world [2], which imposed a heavy burden on global health care system and services. Meanwhile, the annual outbreak of influenza epidemics remains a significant public health threat and results in substantial morbidity and mortality globally [3]. Influenza infection usually peaks in winter seasons [4], circulating in parallel with COVID-19, which could pose burden on the prevention and treatment of both diseases.

Various epidemiology studies have reported pregnancy as a risk factor for influenza infection [5, 6]. Due to the physiological and immunological changes during pregnancy, pregnant women are proved to be more susceptibility to influenza infection and serious related outcomes [7, 8]. In 2012, WHO recommended pregnant women as the prior population to get influenza vaccination [9]. Despite increasing recommendations to vaccinate pregnant women in many countries, influenza vaccination coverage around the world remained suboptimal. Research has found that the coverage of influenza vaccination among pregnant women in recent years was far lower than the targeted uptake rate in America [10]. According to national surveys in multiple European countries, the median influenza vaccine coverage rate among pregnant women was merely 9% from 2008/2009 to 2014/2015 [11], indicating that more attention and publicity should be given from related organizations to raise pregnant women’s awareness of their vulnerability to seasonal influenza and the significance of influenza vaccination, while understanding the causes of their vaccine hesitancy.

Influenza vaccination serves as an effective way to prevent pregnant women from seasonal influenza infection [12, 13]. Furthermore, in the context of COVID-19 pandemic, seasonal influenza vaccination is believed to reduce the risk of other respiratory disease infections such as COVID-19, for minimizing potential cross infection acquired by hospital visits due to influenza infection [14]. Influenza vaccination at high coverage also decreases influenza hospital admissions, therefore reduces the uptake of medical resource, alleviates social health care systems from the significant pressure of dealing with both COVID-19 and influenza at the same time [15]. What’s more, considering the similar symptoms that COVID-19 and seasonal influenza present, influenza vaccination adds benefit to the specificity and accuracy of syndromic COVID-19 surveillance [14].

Present studies suggest that pregnant women may be particularly vulnerable to COVID-19 infection [16, 17]. Nonetheless, current clinical trials that evaluate vaccine candidates do not include pregnant women, resulting in insufficient data on the safety and effectiveness of COVID-19 vaccines to this special population. Thus, accounting the addition health benefit of influenza vaccination in the context of COVID-19 mentioning above, along with pregnant women’s vulnerability to influenza, it is of great significance for pregnant women to receive timely influenza vaccination during influenza seasons during COVID-19 pandemic period. However, due to pregnant women’s lack of relevant knowledge [18, 19], fear of potential adverse events [20] uncertainty regarding the safety of vaccine [18] and insufficient recommendations [21, 22], pregnant women are often unwilling to receive seasonal influenza vaccination. Influenza hesitancy serves as an obstacle to vaccination. Hence, recognizing the acceptance and knowledge of influenza vaccination among pregnant women, as well as the causes of vaccine hesitancy is essential for relevant organizations and health care workers to take targeting measures to address hesitancy and improve vaccine uptake, especially during COVID-19 pandemic period when pregnant women’s willingness of influenza vaccination may be influenced.

Owing to the scarceness of research on the acceptance, knowledge and barriers of influenza vaccination among pregnant women in the context of COVID-19 pandemic, we conducted a hospital-based multi-center cross-sectional study among pregnant women in mainland China based on Health Belief Model (HBM) to evaluate the acceptance of influenza vaccination and associated factors among pregnant women in the context of COVID-19 pandemic. Our secondary objective is to provide evidence for certain tailored measures and strategies to expand influenza vaccination coverage among pregnant women, so as to help reduce the risk of co-infection and alleviate the burden of health care system for treating both COVID-19 patients and influenza patients.

## Methods

### Study design and participants

We conducted a multi-center cross-sectional survey among a sample of pregnant women in mainland China. A multistage sampling approach was applied to select participants. The initial sampling framework was based on the guidelines of National Bureau of Statistics of China, which divided mainland China into three (eastern, central and western) regions. Five provinces were randomly selected from the three regions in China, namely Beijing (eastern), Hebei (eastern), Hubei (central), Anhui (central), and Yunnan (western). In the second stage of sampling, we selected a convenience sample of ten hospitals from the five provinces of China, including Tongzhou Maternal and Child Health Hospital (Beijing), Haidian Maternal and Child Health Hospital (Beijing), Shexian Maternal and Child Health Hospital (Hebei), Songzi Maternal and Child Health Hospital (Hubei), Qianjiang Maternal and Child Health Hospital (Hubei), Jinmen People’s hospital (Hubei), Mingguang Maternal and Child Health Hospital (Anhui), the Second Affiliated Hospital of Kunming Medical University (Yunnan), Qujing Maternal and Child Health Hospital (Yunnan), and Xuanwei Maternal and Child Health Hospital (Yunnan). In the third stage, pregnant women who attended antenatal clinics of the selected ten hospitals during November 13, 2020 to December 31, 2020 were approached for study participation. Inclusion criteria were 1) pregnant women who aged 18 years or above; 2) pregnant women who presented to antenatal clinics in the selected hospitals from 13 November 2020 to 31 December 2020; 3) providing voluntary consent to participate in the study.

PASS software 14.0 (NCSS LLC., Kaysville, U.T., USA) was used to calculate the sample size. According to extant literature, the rate of influenza vaccination among pregnant women in Taiwan province in our country was 64.5% [18]. Set the alpha as 0.05, the confidence interval width as 0.1p, the sample size was 352 for each province when using the exact (Clopper-Pearson) method for calculation. Therefore, the sample size was 1760 in five provinces. Considering 15% non-response rate, the sample size of this study was planned to be 2200 participants.

The study was conducted in accordance with the Helsinki Declaration and was approved by the Ethical Committee of Peking University Third Hospital. Informed consent was obtained from all participants who are included in the present study.

### Data collection

Our structured questionnaire framework was based on the Health Belief Model [23], a commonly adopted model to investigate participants’ vaccine hesitancy and understand associated factors. Additionally, we constructed and modified our questionnaire (Supplementary file 1) according to previous literature on the willingness of vaccination on influenza among pregnant women with questionnaires of high validity [19, 24, 25]. By conducting the survey, we collected data on sociodemographic characteristics, health status, knowledge of both seasonal influenza infection and influenza vaccine, attitude towards seasonal influenza vaccination, and health beliefs on influenza infection and vaccination among pregnant women. We conducted a pilot test among a convenience sample of 20 pregnant women and the questionnaire showed an adequate internal consistency reliability (Cronbach’s α = 0.81).

### Sociodemographic characteristics and health status

Sociodemographic characteristics consisted of participants’ age group, region, education, occupation, and monthly household income per capita. Health status comprised gravidity, parity, gestational trimester, gestational complications, and prior experience of adverse pregnancy outcomes, chronic disease, and influenza vaccination.

We investigated participants’ prior experience of adverse pregnancy outcomes by asking ‘Do you have the history of any adverse pregnancy outcomes, such as miscarriage, low birth weight, stillbirth, preterm birth or macrosomia?’ and was answered as ‘yes’ or ‘no’. Similarly, the history of chronic disease was asked as ‘have you been diagnosed as having any chronic disease, such as cardiovascular disease, diabetes, hypertension, respiratory diseases, or cancer?’. Gestational complications involved gestational diabetes mellitus, gestational hypertension, gestational thyroid disorder and gestational anemia in this questionnaire.

### Knowledge of influenza infection and influenza vaccine

In our study, knowledge of influenza infection consisted of five aspects, including common symptoms, preventive measures, route of transmission, susceptible population and the seasonality of influenza. Knowledge of influenza vaccine included participants’ knowledge of the appropriate time for influenza vaccination among pregnant women, the effectiveness of influenza vaccine, intervals between vaccinations, and the prior populations for influenza vaccination. All the items can be answered as ‘yes’, ‘no’, or ‘not sure’. We assigned 15 items about knowledge of seasonal influenza infection and 5 items about knowledge of influenza vaccine with a total score of 15 and 5 respectively. A score of 1 represented correct answer while 0 represented wrong answer or ‘not sure’ response. The knowledge score of influenza infection and vaccine were divided into three degrees, with a score from 0 to 4 representing low degree, 5 to 10 representing moderate degree and 11 to 15 representing high degree in the case of knowledge of influenza infection. The score of knowledge of influenza vaccination were equally divided into three groups from 0 to 5, in accordance with the three degrees.

### Attitude towards influenza vaccination

We assessed participants’ attitude towards influenza vaccination by asking participants’ acceptance of seasonal influenza vaccine if offered. If pregnant women responded ‘no’ or ‘not sure’, they were then asked for the causes of their vaccine hesitancy.

### Health beliefs on influenza infection and vaccination

We assessed participants’ vaccine hesitancy and the associated factors based on HBM. The HBM contained five aspects that influence individuals’ health behaviors, namely perceived susceptibility, severity, barriers, benefits and cues to action. According to the assumption of HBM, individuals are more likely to receive influenza vaccination if they perceive that they are susceptible to influenza, the disease is severe, their behavior of vaccination is beneficial, or the barriers are minimal [24]. In addition, recommendations and education from health care workers on vaccination also influence the vaccination behaviors, which reflects cues to action [19]. A total of 12 items related to the HBM were incorporated in the questionnaire, including two on perceived susceptibility to influenza infection for both mother and infant, two on perceived severity of influenza infection for mother and infant, three on perceived barriers of influenza vaccination, three on benefits of influenza vaccination and two on cues to action. Each item was answered on a three-point Likert scale (‘very concerned or agree, ‘moderate concerned or not sure’, ‘not concerned or disagree’) and was assigned as 3, 2, and 1 score, respectively. The participants were divided into three tertiles (low, moderate, high) according to the summed score calculated based on according items for each HBM aspect. The scores of low, moderate and high tertiles were 2 to 3, 4 to 5 and 6 in perceived susceptibility, perceived severity and cues to action, while the scores of low, moderate and high tertiles were 3 to 4, 5 to 7, 8 to 9 in perceived barriers and benefits.

### Data analysis

All the data analyses were conducted by R (version 3.6.3) and SAS (version 9.4). We described the characteristics (sociodemographic characteristics, health status, knowledge factors and health beliefs) of all pregnant women enrolled by frequencies and percentages. The proportions and the 95% confidence interval (CI) of the acceptance of influenza vaccination among participants of different characteristics and responses were calculated. Pearson’s χ^2^ test was used to compare the acceptance of influenza vaccination by the characteristics mentioned above, while Cochran-Armitage test for trend was adopted to examine the trend of proportion of the acceptance of influenza vaccine by characteristics.

We used multivariable logistic regression model with the adjustment for sociodemographic characteristics, health status, knowledge scores on influenza infection and vaccine, and health belief to identify factors associated with the acceptance of influenza vaccine. Adjusted odds ratio (aOR) and its 95%CI for each variable were calculated. Locally weighted scatterplot smoothing regression analysis [26] was used to evaluate the trends in the acceptance of influenza vaccine and the knowledge scores on influenza infection and vaccine. A *p* value of < 0.05 was considered significant in Pearson’s χ^2^ test, Cochran-Armitage test for trend and multivariable logistic regression.

## Results

### Characteristics of the study population

A total of 2568 eligible pregnant women were included in this study (Table 1). Among them, 1516 (59.0%) were 30 years old or below, 798 (31.1%) had a bachelor’s degree or above, 1168 (45.5%) were housewives, and 966 (37.6%) were first pregnant women. 811 (31.6%) pregnant women had a history of adverse pregnancy outcomes, 46 (1.8%) of them had a history of chronic disease and 513 (20.0%) were diagnosed with gestational complications in the current pregnancy. It is notable that only 213 (8.3%) of the participants had a history of seasonal influenza vaccination.

**Table 1:** Acceptance of seasonal influenza vaccination among pregnant women in China by sociodemographic characteristics, medical and knowledge factors (*n* = 2568)

Characteristics	Number (%)	Acceptance of seasonal influenza vaccination		*p* value
		n (%)	95% CI	
**Sociodemographic characteristics**				
Age group				0.13
<=25	466 (18.1)	370 (79.4)	75.6–82.9	
26–30	1050 (40.9)	811 (77.2)	74.6–79.7	
31–35	772 (30.1)	582 (75.4)	72.3–78.3	
36–40	224 (8.7)	163 (72.8)	66.7–78.3	
> 40	56 (2.2)	38 (67.9)	55.0–79.0	
Region				0.01*
Eastern	1266 (49.3)	941 (74.3)	71.9–76.7	
Central	549 (21.4)	420 (76.5)	72.8–79.9	
Western	753 (29.3)	603 (80.1)	77.1–82.8	
Education				< 0.01*
Less than high school	579 (22.5)	429 (74.1)	70.4–77.5	
High school or some college	1191 (46.4)	943 (79.2)	76.8–81.4	
Bachelor ‘s degree	692 (26.9)	522 (75.4)	72.1–78.5	
Postgraduate degree	106 (4.1)	70 (66.0)	56.7–74.5	
Occupation				0.73
Housewife	1168 (45.5)	897 (76.8)	74.3–79.2	
Employed	1400 (54.5)	1067 (76.2)	73.9–78.4	
Monthly household income per capita (RMB)				0.99
<=3000	635 (24.7)	487 (76.7)	73.3–79.9	
3001–5000	841 (32.7)	645 (76.7)	73.7–79.5	
5001–10,000	751 (29.2)	572 (76.2)	73.0–79.1	
> 10,000	341 (13.3)	260 (76.2)	71.5–80.5	
**Health status**				
Gravidity				0.43
1	966 (37.6)	747 (77.3)	74.6–79.9	
> = 2	1602 (62.4)	1217 (76.0)	73.8–78.0	
Parity				0.12
0	1274 (49.6)	991 (77.8)	75.4–80.0	
> = 1	1294 (50.4)	973 (75.2)	72.8–77.5	
Gestational week				< 0.01*
First trimester (1–13 week)	747 (29.1)	538 (72.0)	68.7–75.2	
Second trimester (14–28 week)	725 (28.2)	563 (77.7)	74.5–80.6	
Third trimester (> = 28 week)	1096 (42.7)	863 (78.7)	76.2–81.1	
History of adverse pregnancy outcomes				0.63
Yes	811 (31.6)	625 (77.1)	74.1–79.9	
No	1757 (68.4)	1339 (76.2)	74.2–78.2	
History of chronic disease				0.18
Yes	46 (1.8)	39 (84.8)	72.4–92.9	
No	2522 (98.2)	1925 (76.3)	74.6–78.0	
History of influenza vaccination				< 0.01*
Yes	213 (8.3)	184 (86.4)	81.3–90.5	
No	2355 (91.7)	1780 (75.6)	73.8–77.3	
Gestational complications				0.07
Yes	513 (20.0)	408 (79.5)	75.9–82.9	
No	2055 (80.0)	1556 (75.7)	73.8–77.5	
**Knowledge factors**				
Total knowledge score on seasonal influenza infection				< 0.01*
Low (score 0–4)	175 (6.8)	107 (61.1)	53.8–68.1	
Moderate (score 5–10)	889 (34.6)	621 (69.9)	66.8–72.8	
High (score 11–15)	1504 (58.6)	1236 (82.2)	80.2–84.1	
Total knowledge score on seasonal influenza vaccination				< 0.01*
Low (score 0–1)	1507 (58.7)	1059 (70.3)	67.9–72.5	
Moderate (score 2–3)	907 (35.3)	763 (84.1)	81.6–86.4	
High (score 4–5)	154 (6.0)	142 (92.2)	87.2–95.7	
Total	2568 (100.0)	1964 (76.5)	74.8–78.1	

*p < 0.05

### Acceptance of seasonal influenza vaccine of pregnant women regarding sociodemographic characteristics, health status and knowledge

**Fig. 1:**
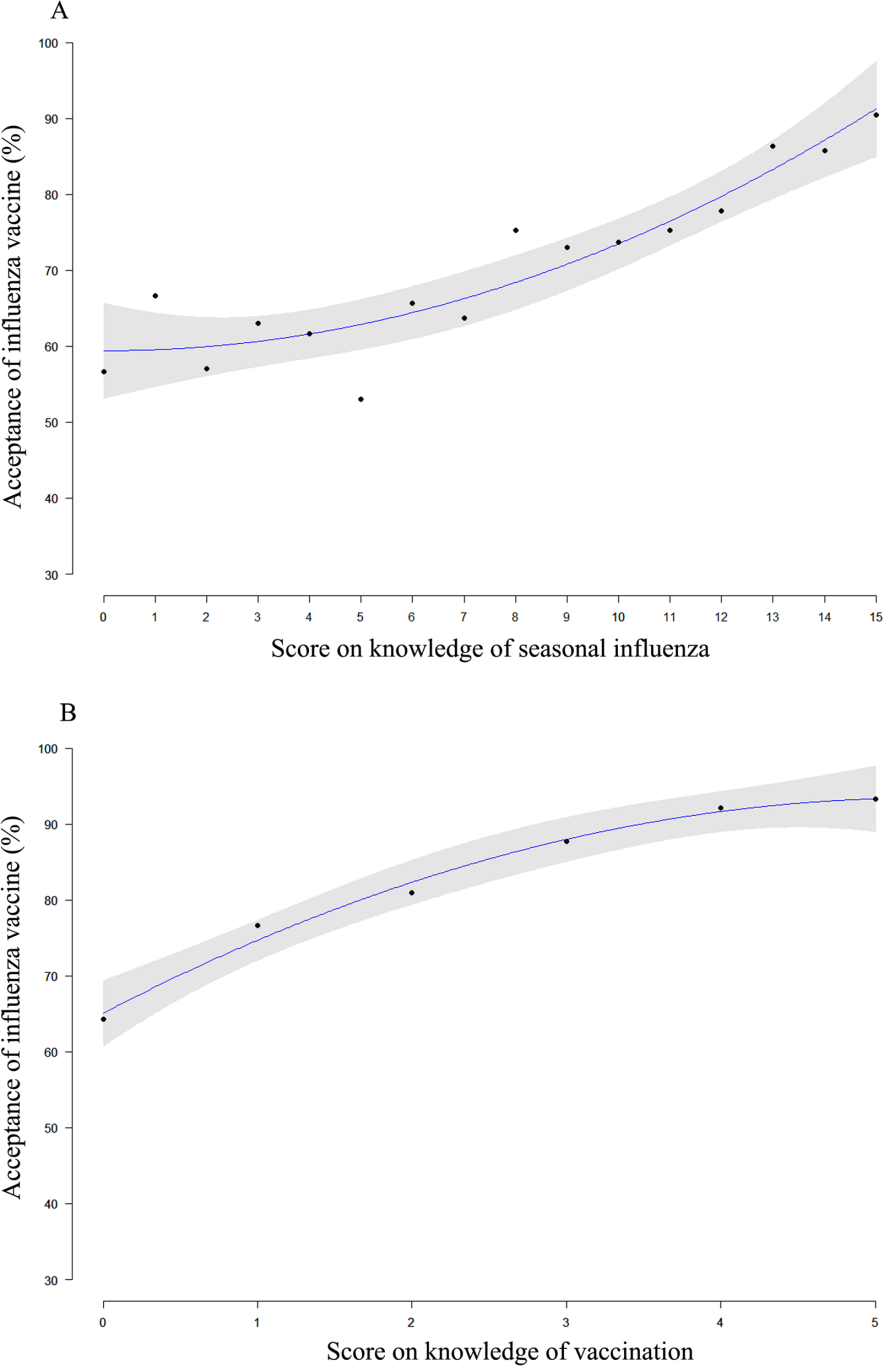
The trends in the acceptance of seasonal influenza vaccination and the total knowledge score on seasonal influenza and vaccine by locally weighted scatterplot smoothing regression analysis: **A** knowledge score on seasonal influenza infection; **B** knowledge score on seasonal influenza vaccine

The total acceptance proportion of seasonal influenza vaccine was 76.5% (95%CI 74.8–78.1%) among all participants. The proportion ranged from high to low in accordance with the increasing age groups, varying from 79.4% in women aged 25 years or below to 67.9% in women aged over 40 years. However, no significant relationship between ages and acceptance rates was observed (*p* = 0.13). In unadjusted model, pregnant women with a lower education, living in western region, currently in the second or third gestational trimester, with gestational complications, and have higher knowledge score on both seasonal influenza infection and vaccine were more prone to accept influenza vaccination (all *p* < 0.05, Table 1). Locally weighted scatterplot smoothing regression analysis indicated that the acceptance proportions of influenza vaccine significantly increased with the increasing knowledge scores on influenza infection and vaccine (*p* < 0.01, Fig. 1).

### Comparison of the acceptance of seasonal influence vaccine in health belief model

**Fig. 2:**
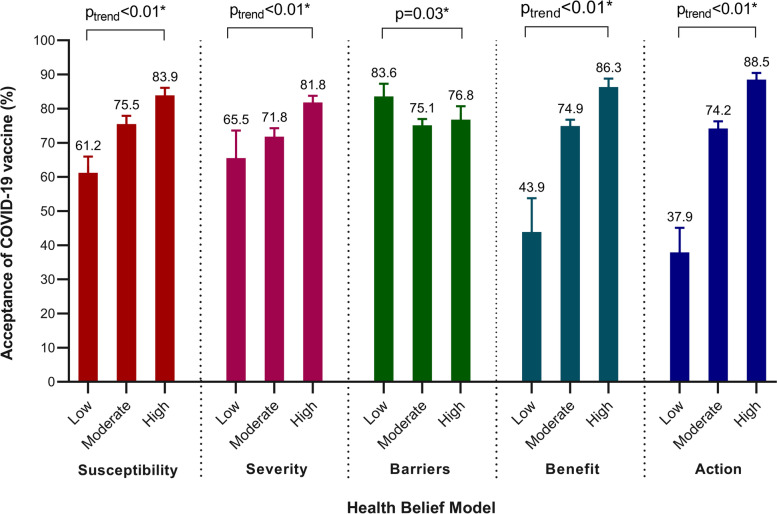
The acceptance of a COVID-19 vaccine by five dimensions of Health Beliefs Model (n = 2568)

Pregnant women who were concerned about themselves infected by seasonal influenza were more likely to accept influenza vaccination (79.1, 95%CI: 77.4–80.8%) than those were not concerned (61.3,95%CI: 56.3–66.1%, *p* < 0.01, Table 2). Similarly, those who were concerned about their unborn babies infected by seasonal influenza had a higher likelihood to accept influenza vaccination (78.5, 95%CI: 76.8–80.1%), compared to the unconcerned ones (60.4, 95%CI: 54.6%-65.9, *p* < 0.01). Pregnant women who perceived that they were more likely to have severe illness if infected by seasonal influenza had greater possibility to accept influenza vaccination (77.1, 95%CI: 75.4–78.8%) than those who had contrary perception (68.3, 95%CI: 61.4–74.6%, *p* < 0.01). In the ‘barriers’ dimension of HBM, pregnant women who believed that seasonal vaccination was not safe during pregnancy or not effective against influenza (safety: 75.3, 95%CI: 73.4–77.0%; effectiveness: 75.1, 95%CI: 73.2–76.8%) had lower levels of acceptance than those who disagreed (safety: 85.3, 95%CI: 81.1–88.9%, *p* < 0.01; effectiveness: 84.3, 95%CI: 80.5–87.6%, p < 0.01). In the ‘benefits’ dimension, those who agreed with the benefit of vaccination to themselves(77.6, 95%CI: 75.9–79.2%), their fetus and new born babies (77.9, 95%CI: 76.2–79.5%) and the protection for babies during first month of life (77.5, 95%CI: 75.8–79.1%) had higher levels of acceptance than those who disagreed (all *p* < 0.01; benefit pregnant women themselves: 51.8, 95%CI: 42.6–60.8%; benefit their fetus and new born babies: 53.6, 95%CI: 45.7–61.5%; protect babies for one month: 57.5, 95%CI: 48.8–65.8%). Pregnant women who received cues to action from physicians (78.9, 95%CI: 77.3–80.5%) or family members (79.2, 95%CI: 77.6–80.8%) were more likely to accept influenza vaccination than those who didn’t (all *p* < 0.01; physicians: 38.1, 95%CI: 30.7–45.9%; family members: 43.4, 95%CI: 36.7–50.4%). The acceptance proportions of influenza vaccine among participants were significantly higher in those with high level of perceived susceptibility, severity, benefits and cues to action (all p < 0.01), while it was significantly lower in pregnant women with higher level of perceived barriers of vaccination (*p* = 0.03, Fig. 2).

**Table 2:** Comparison of the acceptance of seasonal influenza vaccination by health belief factors in the health belief model (n = 2568)

Dimensions of Health Belief Model	Item	Response	Number (%)	Acceptance of seasonal influenza vaccination		*p* value
				n (%)	95% CI	
Perceived susceptibility	Are you concerned about getting seasonal influenza	Not concerned	380 (14.8)	233 (61.3)	56.3–66.1	< 0.01*
		Concerned	2188 (85.2)	1731 (79.1)	77.4–80.8	
	Are you concerned about unborn baby getting seasonal influenza	Not concerned	285 (11.1)	172 (60.4)	54.6–65.9	< 0.01*
		Concerned	2283 (88.9)	1792 (78.5)	76.8–80.1	
Perceived severity	If a pregnant woman gets seasonal influenza, she is more likely to have severe illness	Disagree	189 (7.4)	129 (68.3)	61.4–74.6	< 0.01*
		Agree	2379 (92.6)	1835 (77.1)	75.4–78.8	
	If a pregnant woman gets seasonal influenza, the illness could harm her unborn baby	Disagree	78 (3.0)	54 (69.2)	58.4–78.6	0.13
		Agree	2490 (97.0)	1910 (76.7)	75.0–78.3	
Perceived barriers	Seasonal influenza vaccination can cause a person to get sick with seasonal influenza	Disagree	695 (27.1)	550 (79.1)	76.0–82.0	0.05
		Agree	1873 (72.9)	1414 (75.5)	73.5–77.4	
	Seasonal influenza vaccination is not safe during pregnancy	Disagree	313 (12.2)	267 (85.3)	81.1–88.9	< 0.01*
		Agree	2255 (87.8)	1697 (75.3)	73.4–77.0	
	Vaccine is not an effective way to prevent a pregnant woman from getting seasonal influenza	Disagree	395 (15.4)	333 (84.3)	80.5–87.6	< 0.01*
		Agree	2173 (84.6)	1631 (75.1)	73.2–76.8	
Perceived benefits	Giving vaccine to a pregnant woman will benefit her fetus and new born baby	Disagree	151 (5.9)	81 (53.6)	45.7–61.5	< 0.01*
		Agree	2417 (94.1)	1883 (77.9)	76.2–79.5	
	Getting vaccine during pregnancy is a benefit for the pregnant woman	Disagree	114 (4.4)	59 (51.8)	42.6–60.8	< 0.01*
		Agree	2454 (95.6)	1905 (77.6)	75.9–79.2	
	Vaccine could protect the baby during the first month of life	Disagree	127 (4.9)	73 (57.5)	48.8–65.8	< 0.01*
		Agree	2441 (95.1)	1891 (77.5)	75.8–79.1	
Cues to action	If physician recommended vaccine, I would get vaccinated	Disagree	155 (6.0)	59 (38.1)	30.7–45.9	< 0.01*
		Agree	2413 (94.0)	1905 (78.9)	77.3–80.5	
	If family members recommended vaccine, I would get vaccinated	Disagree	198 (7.7)	86 (43.4)	36.7–50.4	< 0.01*
		Agree	2370 (92.3)	1878 (79.2)	77.6–80.8	

Response of “not sure” was combined into “disagree” and “very concerned “or “moderate concerned” was combined into “concerned” in this table

*p < 0.05

### Factors associated with the acceptance of influenza vaccine

Factors that significantly associated with the acceptance of influenza vaccine in the multivariable logistic regression model (Table 3) were western region (aOR = 1.40, 95% CI: 1.08–1.82), bachelor’s degree (aOR = 0.48, 95% CI: 0.29–0.80), having the history of influenza vaccination (aOR = 1.81, 95% CI: 1.18–2.80), high knowledge score on influenza infection (aOR = 2.21, 95% CI: 1.51–3.23), moderate or high knowledge score on influenza vaccination (moderate: aOR = 1.64, 95%CI:1.28–2.10; high: aOR = 2.63, 95%CI:1.38–5.00), moderate or high level of perceived susceptibility (moderate: aOR = 1.68, 95%CI: 1.28–2.21; high: aOR = 2.48, 95%CI: 1.83–3.35), low or moderate level of perceived barriers (low: aOR = 1.76, 95%CI: 1.29–2.41; moderate: aOR = 2.69, 95%CI: 1.73–4.19), moderate or high level of perceived benefit (moderate: aOR = 1.82, 95%CI: 1.10–3.02; high: aOR = 1.90, 95%CI:1.06–3.41), and moderate or high level of cues to action (moderate: aOR = 4.22, 95%CI: 2.92–6.11; high: aOR = 8.27, 95%CI: 5.35–12.77).

**Table 3:** Factors related with the acceptance of seasonal influenza vaccination (*n* = 1392)

Characteristics	Multivariate model	
	Adjusted OR (95% CI)	***p*** value
**Sociodemographic characteristics**		
Region		
Eastern	1 (Reference)	
Central	0.90 (0.69–1.19)	0.47
Western	1.40 (1.08–1.82)	0.01*
Education		
Less than high school	1.17 (0.90–1.52)	0.24
High school or some college	0.84 (0.62–1.13)	0.24
Bachelor’s degree	0.48 (0.29–0.80)	0.01*
Postgraduate degree	1 (Reference)	
Gestational week		
First trimester (1–13 week)	1 (Reference)	
Second trimester (14–28 week)	1.15 (0.88–1.51)	0.30
Third trimester (> = 28 week)	1.27 (0.98–1.65)	0.07
History of influenza vaccination		
Yes	1.81 (1.18–2.80)	0.01*
No	1 (Reference)	
**Knowledge on seasonal influenza and vaccine**		
Total knowledge score on seasonal influenza infection		
Low	1 (Reference)	
Moderate	1.42 (0.99–2.05)	0.06
High	2.21 (1.51–3.23)	< 0.01*
Total knowledge score on seasonal influenza vaccine		
Low	1 (Reference)	
Moderate	1.64 (1.28–2.10)	< 0.01*
High	2.63 (1.38–5.00)	< 0.01*
**Health belief factors**		
Perceived susceptibility		
Low	1 (Reference)	
Moderate	1.68 (1.28–2.21)	< 0.01*
High	2.48 (1.83–3.35)	< 0.01*
Perceived severity		
Low	1 (Reference)	
Moderate	1.02 (1.66–0.62)	0.94
High	1.11 (1.78–0.69)	0.67
Perceived barriers		
Low	1.76 (1.29–2.41)	< 0.01*
Moderate	2.69 (1.73–4.19)	< 0.01*
High	1 (Reference)	
Perceived benefit		
Low	1 (Reference)	
Moderate	1.82 (1.10–3.02)	0.02*
High	1.90 (1.06–3.41)	0.03*
Cues to action		
Low	1 (Reference)	
Moderate	4.22 (2.92–6.11)	< 0.01*
High	8.27 (5.35–12.77)	< 0.01*

*p < 0.05

### Reasons for having no intention to receive seasonal influenza vaccine

**Fig. 3:**
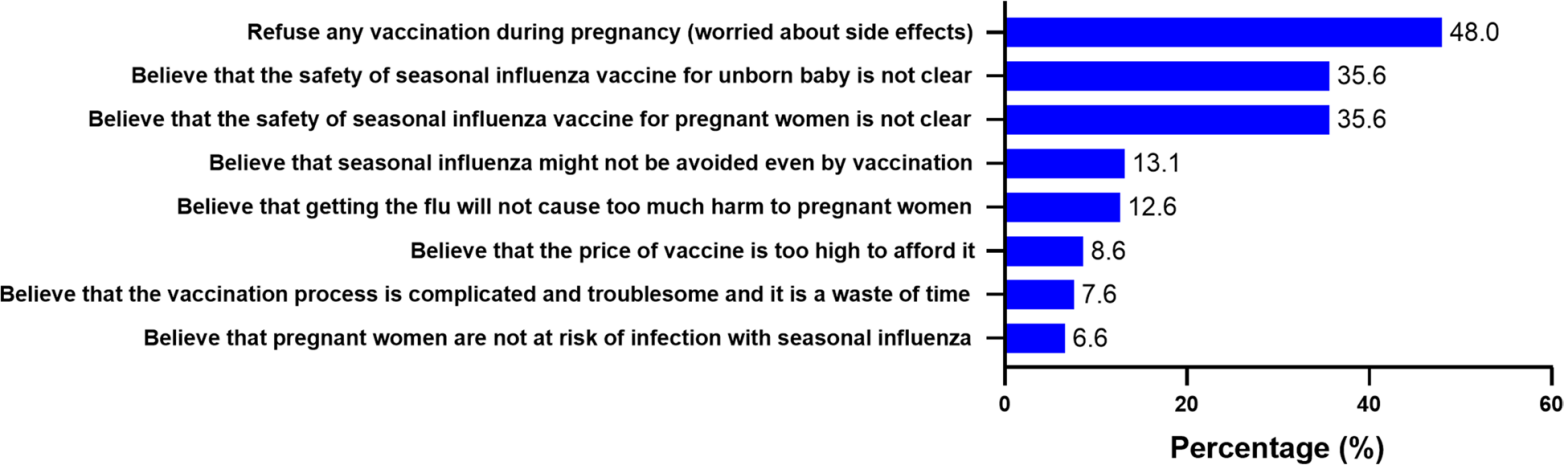
Reasons for pregnant women’s influenza vaccination hesitancy (*n* = 604)

Among the 2568 participants, 604 (23.5%) pregnant women had vaccine hesitancy, responding ‘no’ or ‘not sure’ regarding the intention of receiving influenza vaccine if offered. 48.0% of them refused any vaccination during pregnancy, for worrying about any side effect. 35.6% of them believed that the safety of seasonal influenza vaccine for both their unborn babies and themselves remained unclear. Furthermore, the time-consuming process of vaccination, high price of vaccine, belief of themselves having little risk of infection, believing influenza would not cause severe damage to their health and the uncertainty of vaccine effectiveness were also reasons for pregnant women’s influenza vaccine hesitancy (Fig. 3).

## Discussion

To our best knowledge, this is the first study to evaluate the acceptance of seasonal influenza vaccination and associated factors among pregnant women in the context of COVID-19 pandemic. We conducted a multi-center hospital-based cross-sectional study in mainland China based on the Health Belief Model, in order to have a better understanding of the acceptance, knowledge and barriers of influenza vaccination among pregnant women, provide evidence for healthcare workers and policy makers to identify potential barriers and take tailored measures to expand influenza vaccination coverage during the COVID-19 pandemic period. Our study found that the acceptance of influenza vaccination in the context of COVID-19 pandemic among pregnant women was 76.5% (95%CI: 74.8–78.1%). Pregnant women in western region, with low education level, having prior experience of influenza vaccination, with a high level of knowledge of influenza infection and vaccine, high levels of perceived susceptibility, benefits and cues to action, low level of perceived barriers are prone to accept seasonal influenza vaccination.

Previous researches have shown that pregnant women were not only susceptible to influenza infection, pregnant women with seasonal influenza infection are also at elevated risk of severe complications and adverse pregnancy outcomes such as fetal loss, higher rates of hospitalization, preterm birth, and maternal death [27–29]. In addition, pregnant women are believed to be more susceptible to respiratory pathogens, resulting in their susceptibility to COVID-19 infection compared to the general population [17]. Recent studies have observed significant associations between COVID-19 infection and severe pregnancy outcomes including fetal death, preterm birth, preeclampsia, and cesarean [30, 31]. However, as evidence on the association between COVID-19 and adverse pregnancy outcomes is still emerging, more studies and data are needed to further investigate the effect of COVID-19 on pregnancy outcomes, maternal complications and vertical transmission.

Due to the similar route of transmission and time of onset between COVID-19 and seasonal influenza, individuals are at higher risk of being co-infected by COVID-19 and influenza in the context of COVID-19 pandemic. Although case series of patients co-infected with influenza and COVID-19 did not observe significant difference in the clinical characteristics between patients co-infected and those of single COVID-19 infection, the symptoms and outcomes of the co-infected were no better than those infected with COVID-19 [32, 33]. Yue and his college found that patients co-infected with coronavirus 2 and influenza B virus had a higher rate of presenting poor prognosis than patients who were infected by COVID-19 only [34]. Additional cases and clinical information are warranted for a conclusive and comprehensive conclusion on the effect of co-infection on individual’s health, especially among pregnant women.

Influenza vaccination is believed by many scholars to be beneficial for the prevention of COVID-19, for reducing the risk of co-infection during individual’s visits to hospitals [14, 15]. Data has demonstrated that influenza vaccination may also have impact on the susceptibility and clinical outcomes of COVID-19. A meta-analysis that included 12 observational studies suggested that influenza vaccination was associated with a lower risk of COVID-19 infection [35]. A protective effect against death and hospitalization was observed for COVID-19 patients aged above 65 who received influenza vaccination in parallel with the outbreak of COVID-19, although no significant effect was observed in the overall patients [36]. In Brazil, patients with COVID-19 infection who recently received influenza vaccination had significantly better clinical outcomes (the need of intensive care treatment, invasive respiratory support requirement and death) than non-vaccinated patients. The protective effect was more significant in younger patients and those who received seasonal influenza vaccination after the onset of symptoms [37]. Arokiaraj and his college [38] collected data from 34 countries worldwide and found that influenza vaccination correlated with lower morbidity, mortality and case incidence in COVID-19. The protective effect of influenza vaccination on COVID-19 may be explained by trained immune responses triggered by vaccine. It is thus advisable to promote large-scale influenza vaccination, especially in populations at high risk of infection and severe complications.

In our study, we estimated that the acceptance of influenza infection among pregnant women in the context of COVID-19 was 76.5%, which was slightly higher than that of a survey carried out among the general population in Italy during the COVID-19 pandemic. In the survey conducted in Italy among general population, 74.8% of the participants valued influenza vaccination positively and believed that it should be mandatory [39]. This may result from pregnant women’s higher level of perceived susceptibility and severity than the general population, for fearing that influenza infection during the COVID-19 period was harmful to both their health and their fetus. The acceptance rate of our study during the COVID-19 pandemic was also higher than the acceptance rate of influenza infection among pregnant women in Taiwan in 2018 (64.5%) [18] and that in Thailand in 2015 (41.8%) [24], indicating that women may address higher attention and awareness to their and their fetus’ health during the global pandemic that has caused global public health emergency and massive impact on social economy. Only 8.3% of the pregnant women in our survey had a history of influenza vaccination. The vaccination coverage in the population included in our study was lower than that among pregnant women in America [40] (53.6%), Nicaragua [41] (41.8%), Italy (9.7%) [42] in recent years, but was higher than that in France [43] (7.4%) in 2020. The universal vaccination coverage in pregnant women still awaits to be improved, especially during the COVID-19 pandemic period that would largely benefit from seasonal influenza vaccination as mentioned above.

As indicated in our study, pregnant women who live in western region, with low education level, having prior experience of influenza vaccination and with a high level of knowledge of influenza infection and vaccine are more prone to accept influenza vaccination. The result that previous influenza vaccination history positively associates with the willingness of accepting influenza vaccination was consistent with previous studies [18, 21, 44], suggesting that prior vaccination experience may add to pregnant women’s confidence and reduces their uncertainty and doubts of vaccination. It has also been reported in previous studies that pregnant women of high level of knowledge of influenza and vaccine have higher intentions to receive vaccination during pregnancy [19, 24, 44]. Sufficient knowledge of the effects of influenza on themselves and their babies, as well as the benefits of vaccine could mitigate their concerns and improve their willingness of vaccination. However, we also found that pregnant women with high level of education have a greater likelihood of vaccination refusal, which seemed contradictory to the effect of high knowledge on vaccination acceptance. We assumed that pregnant women with high level of education had more access to various information about influenza and vaccine, which may increase the likelihood for them to receive inaccurate, biased or extreme information, leading to their potential misunderstanding of the safety of vaccine. Therefore, it’s high time that the government and relevant organizations should provide more timely information of influenza and vaccine and enhance publicity and professional health education, in order to reduce public concerns and improve public knowledge about vaccine safety and effectiveness.

Regarding the health belief of pregnant women towards influenza vaccination, high levels of perceived susceptibility, benefits and cues to action are positively associated with the willingness of vaccination, while high level of perceived barriers are negatively associated with it. The results suggested that more information about pregnant women’s susceptibility to influenza infection and severe complications should be widely provided and underscored. Meanwhile, we should give full publicity to the benefits brought by influenza vaccine particularly in the context of COVID-19 pandemic, that influenza vaccine is not only beneficial to protect pregnant women and their fetus from infection and harm, but also have health and economic benefits for COVID-19 pandemic [14]. It is also advisable for health care workers to take measures to spread professional knowledge and explain pregnant women’s potential questions and concerns about influenza vaccine, so that perceived barriers and lack of confidence may be alleviated. Multiple studies have highlighted the significance of cues to action [20, 21, 25, 44], suggesting that recommendations from family members, physicians and friends serve as motivation for vaccination. Therefore, vaccine campaigns and publicity can also target family members and health care providers of the high-risk population.

The study has several limitations. First, participants were recrutied only if they attended antenatal clinics. Thus, the results may not be generalizable to all pregnant women which include those who didn’t attend antenatal clinics. Second, the specific effect of COVID-19 pandemic on pregnant women’s willingness of influenza vaccinaiton was not fully evaluated in our study. More in-depth studies are warranted to further explore the effect of COVID-19 pandemic on influenza vaccination. Third, the study centred on the accpetance and its related factors of influenza vaccination in the context of COVID-19 pandemic, which did not inevitably lead to the actual behavior of influenza vaccination. More statistics regarding influenza vaccination coverage among pregnant women during the COVID-19 period are needed. Last, recall bias is possible.

## Conclusions

In this multi-center cross-sectional study, the acceptance of influenza vaccination in the context of COVID-19 pandemic among pregnant women was 76.5%. Western region, low education level, prior experience of influenza vaccination, high level of knowledge of influenza infection and vaccine, high levels of perceived susceptibility, benefits and cues to action and low level of perceived barriers are significantly associated with the acceptance of seasonal influenza vaccine. Our findings suggest that tailored strategies and publicity for influenza vaccination in the context of COVID-19 pandemic are urgently needed to improve their comprehensive knowledge about influenza and vaccines and inform them of the safety, effectiveness and benefits of influenza vaccines to reduce their concerns. Health care workers and family members of pregnant women should also be targeted to recommend influenza vaccines to pregnant women.

## Supplementary Information

**Additional file 1: Supplemental file 1**. Questionnaire.

## Data Availability

The datasets used and/or analysed during the current study are available from the corresponding author on reasonable request.

## Abbreviations

COVID-19: Coronavirus disease
