# Supplementary material for: Acceptance of seasonal influenza vaccination and associated factors among pregnant women in the context of COVID-19 pandemic in China: a multi-center cross-sectional study based on health belief model
Source: BMC Pregnancy Childbirth. 2021 Nov 3;21:745. doi: 10.1186/s12884-021-04224-3 (PMC8564270; doi:10.1186/s12884-021-04224-3)
Supplement: Supplementary file 1 — Additional file 1: Supplemental file 1. Questionnaire. [file 12884_2021_4224_MOESM1_ESM.docx]

**Supplemental file 1.**

**Questionnaire**

**Title: Acceptance of seasonal influenza vaccination and associated factors among pregnant women in the context of COVID-19 pandemic in China: A multi-center cross-sectional study based on Health Belief Model**

**Authors:** Ruitong Wang, Liyuan Tao, Na Han, Jihong Liu, Chuanxiang Yuan, Lixia Deng, Chunhua Han, Fenglan Sun, Liqun Chi, Min Liu, Jue Liu

**Correspondence to:** Dr. Jue Liu, Department of Epidemiology and Biostatistics, School of Public Health, Peking University, Beijing 100191, China. (Tel: 86-10-8 2801528-316, Fax: 86-10-8 2805146, Email: jueliu@bjmu.edu.cn)

**A questionnaire about the acceptance of influenza vaccination among pregnant women**

**I. Sociodemographic characteristics and health status**

1. Which age group do you belong to?

A. <=25

B. 26-30

C. 31-35

D. 36-40

E. >40

2. What is your level of education?

A. Less than high school

B. High school or some college

C. Bachelor ‘s degree

D. Postgraduate degree

3. Which is your occupation?

| A. Housewife B. Employed |
| --- |

4. Which is your monthly household income per capita?

| A. <=3000 B. 3001-5000 C. 5001-10000 D. >10000 |
| --- |
|  |

5. What is your current gestational trimester?

| A. First trimester (1-13 week) |
| --- |
| B. Second trimester (14-28 week) |
| C. Third trimester (>=28 week) |

6. This is your ___ pregnancy.

| A. first |
| --- |
| B. second or more |
|  |

7. This is your ____ child.

| A. first |
| --- |
| B. second or more |

8. Do you have the history of any adverse pregnancy outcomes, such as miscarriage, low birth weight, stillbirth, preterm birth or macrosomia?

A. Yes B. No

9. Have you received influenza vaccination before?

A. Yes B. No

10. When was your last influenza vaccination? (if answered ‘Yes’ in question 9 above)

_________________________________

11. Have you been diagnosed as having any chronic disease, such as cardiovascular disease, diabetes, hypertension, respiratory diseases, or cancer?

A. Yes B. No

12. Do you have any gestational complications, such as gestational diabetes mellitus, gestational hypertension, gestational thyroid disorder or gestational anemia?

| A. Yes B. No |
| --- |

**II. Attitude towards influenza vaccination**

13. Are you willing to receive influenza vaccination?

A. Yes B. Not sure C. No

14. Which of the following are the reasons why you are unwilling to receive influenza vaccination? (if answered ‘No’ in question 13 above) (Multiple choice)

A. Pregnant women are not at risk of infection with seasonal influenza.

B. The vaccination process is complicated and troublesome and it is a waste of time.

C. The price of vaccine is too high to afford it.

D. Getting the flu will not cause too much harm to pregnant women.

E. Seasonal influenza might not be avoided even by vaccination.

F. The safety of seasonal influenza vaccine for pregnant women is not clear.

G. The safety of seasonal influenza vaccine for unborn baby is not clear.

H. Refuse any vaccination during pregnancy (worried about side effects).

I. Other reasons: _________________________________

**III.** **Knowledge of influenza infection and influenza vaccine**

15. Which of the following do you think are the common symptoms of influenza.

| A. Fever |
| --- |
| B. Diarrhea |
| C. Muscle soreness |
| D. Sore throat |
| E. Vomiting |
| F. Headache |
| G. Skin rash |
| H. Chest pain |
| I. Not clear |

16. Which of the following do you think are the methods of preventing influenza.

| A. When coughing or sneezing, cover your mouth and nose with tissue, towel, etc. |
| --- |
| B. Wash hands more often. |
| C. Have a balanced diet. |
| D. Obtain moderate exercise and adequate rest. |
| E. Wear a mask. |
| F. Receive influenza vaccines. |
| G. Not clear |

17. Which of the following do you think are the routes of influenza transmission?

| A. Via respiratory droplets |
| --- |
| B. Via oral or eye mucosal contact |
| C. Via infected mosquitoes |
| D. Not clear |

18. Which of the following gestational trimester do you think pregnant women can receive influenza vaccines.

| A. First trimester (1-13 week) |
| --- |
| B. Second trimester (14-28 week) |
| C. Third trimester (>=28 week) |
| D. In any gestational trimester |
| E. Not clear |

19. Do you think the following statements about influenza infection and influenza vaccine are correct?

|  | Yes | Not sure | No |
| --- | --- | --- | --- |
| (1) Influenza vaccination is the most effective way to prevent influenza. | ○ | ○ | ○ |
| (2) Influenza has a seasonal pattern. | ○ | ○ | ○ |
| (3) People are generally susceptible to influenza infection. | ○ | ○ | ○ |
| (4) Pregnant women are susceptible to influenza infection. | ○ | ○ | ○ |
| (5) Pregnant women who receive influenza vaccination can prevent their babies from influenza infection within 6 months old. | ○ | ○ | ○ |
| (6) There is no need for people to receive influenza vaccination again before the coming of the next influenza season if they have been vaccinated in the previous season. | ○ | ○ | ○ |
| (7) Pregnant women are listed as the priority population to receive influenza vaccination in China. | ○ | ○ | ○ |

**IV. Health beliefs on influenza infection and vaccination**

20. Are you concerned about getting seasonal influenza？

A. Very concerned B. Moderate concerned C. Not concerned

21. Are you concerned about the unborn baby getting seasonal influenza？

A. Very concerned B. Moderate concerned C. Not concerned

22. Do you agree with the following statements?

|  | Agree | Not sure | Disagree |
| --- | --- | --- | --- |
| (1) If a pregnant woman gets seasonal influenza, she is more likely to have severe illness. | ○ | ○ | ○ |
| (2) If a pregnant woman gets seasonal influenza, the illness could harm her unborn baby. | ○ | ○ | ○ |
| (3) Seasonal influenza vaccination can cause a person to get sick with seasonal influenza. | ○ | ○ | ○ |
| (4) Seasonal influenza vaccination is not safe during pregnancy. | ○ | ○ | ○ |
| (5) Vaccine is not an effective way to prevent a pregnant woman from getting seasonal influenza. | ○ | ○ | ○ |
| (6) Giving vaccine to a pregnant woman will benefit her fetus and new born baby. | ○ | ○ | ○ |
| (7) Getting vaccine during pregnancy is a benefit for the pregnant woman. | ○ | ○ | ○ |
| (8) Vaccine could protect the baby during the first month of life. | ○ | ○ | ○ |
| (9) If physician recommended vaccine, I would get vaccinated. | ○ | ○ | ○ |
| (10) If family members recommended vaccine, I would get vaccinated | ○ | ○ | ○ |
